# Novel Synthesis of Sensitive Cu-ZnO Nanorod–Based Sensor for Hydrogen Peroxide Sensing

**DOI:** 10.3389/fchem.2022.932985

**Published:** 2022-07-06

**Authors:** Muhammad Arsalan, Imram Saddique, Miao Baoji, Azka Awais, Ilyas Khan, Mohamed A. Shamseldin, Sadok Mehrez

**Affiliations:** ^1^ Henan International Joint Laboratory of Nano-Photoelectric Magnetic Materials, Henan University of Technology, Zhengzhou, China; ^2^ Office of Research Innovation and Commercialization, University of Management and Technology, Lahore, Pakistan; ^3^ Department of Mathematics, University of Management and Technology, Lahore, Pakistan; ^4^ Department of Mathematics, College of Science Al-Zulfi, Majmaah University, Al-Majmaah, Saudi Arabia; ^5^ Mechanical Engineering, Faculty of Engineering and Technology, Future University in Egypt, New Cairo, Egypt; ^6^ Department of Mechanical Engineering, College of Engineering at Al Kharj, Prince Sattam Bin Abdulaziz University, Al Kharj, Saudi Arabia; ^7^ Department of Mechanical Engineering, University of Tunis El Manar, ENIT, Tunis, Tunisia

**Keywords:** hydrothermal method, Cu-ZnO nanorods, electrochemical sensor, H_2_O_2_ detection, sensing

## Abstract

We aimed to synthesize sensitive electrochemical sensors for hydrogen peroxide sensing by using zinc oxide nanorods grown on a fluorine-doped tin oxide electrode by using the facial hydrothermal method. It was essential to keep the surface morphology of the material (nanorods structure); due to its large surface area, the concerned material has enhanced detection ability toward the analyte. The work presents a non-enzymatic H_2_O_2_ sensor using vertically grown zinc oxide nanorods on the electrode (FTO) surfaces with Cu nanoparticles deposited on zinc oxide nanorods to enhance the activity. Scanning electron microscopy (SEM), X-ray photoelectron spectroscopy (XPS), energy-dispersive X-Ray (EDX), X-ray diffraction (XRD), and electrochemical methods were used to characterize copper–zinc oxide nanorods. In addition to the high surface area, the hexagonal Cu-ZnO nanorods exhibited enhanced electrochemical features of H_2_O_2_ oxidation. Nanorods made from Cu-ZnO exhibit highly efficient sensitivity of 3415 μAmM^−1^cm^−2^ low detection limits (LODs) of 0.16 μM and extremely wide linear ranges (0.001–11 mM). In addition, copper-zinc oxide nanorods demonstrated decent reproducibility, repeatability, stability, and selectivity after being used for H_2_O_2_ sensing in water samples with an RSD value of 3.83%. Cu nanoparticles decorated on ZnO nanorods demonstrate excellent potential for the detection of hydrogen peroxide, providing a new way to prepare hydrogen peroxide detecting devices.

## Introduction

Hydrogen peroxide (H_2_O_2_), a reactive oxygen species (ROS) formed by biological processes, is an important biomolecule found in environmental and biological systems and has been used in a wide range of fields such as green energy (fuel cells), medicine (Asif et al., 2022a), food industry, and environmental protection (Chen et al., 2018). In contrast, several studies have indicated that excessive concentrations of H_2_O_2_ adversely affect the body’s normal physiological functions and can cause several diseases in a living organism, such as cardiovascular disease, Alzheimer’s, Parkinson’s, tumors, and cancer (Arsalan et al., 2020; Wu et al., 2020). It is, therefore, vital for both industrial and academic purposes to be able to quickly, easily, and reliably carry out determination of H_2_O_2_ (Arsalan et al., 2021a). The detection of H_2_O_2_ has been accomplished by a number of analytical techniques, including titrometers, spectrophotometers, fluorescence, phosphorescence, chromatographies, and electrochemical techniques (Li et al., 2016; Waqas et al., 2022). The electrochemical technique is a superior alternative to the other methods because of its inherent sensitivity, wide detection range, rapid response, low cost, and good selectivity (Lin et al., 2010; Aziz et al., 2022a). Materials with metal-oxide properties have proven to be effective in the field of electrochemical sensing (Arsalan et al., 2019; Sooppy Nisar et al., 2019; Awais et al., 2021a; Arsalan et al., 2021b). There are many metal oxides with good performance in the biosensor and chemical sensor fields, including tin oxide, tungsten oxide, copper oxide, titanium dioxide, magnesium oxide, cuprous oxide, and zinc oxide (ZnO) (Khan et al., 2019; Awais et al., 2021b; Awais et al., 2021c; Khan et al., 2022). ZnO is an excellent candidate for applications in such environments due to its good catalytic efficiency, biocompatibility, chemical stability, and nontoxicity. In the past few years, ZnO nanostructures have been used in biosensors to detect urate, glucose, and phenolic compounds.

Electrochemical hydrogen peroxide sensors can be split into two classes based on the electrocatalyst utilized as sensor electrodes. The first group uses non-enzymatic electrocatalysts, while the second group uses enzymes, such as GDH and GOx (Zhang et al., 2012). Enzyme-based hydrogen peroxide sensors were first created by Leland C. Clark, also known as the “Father of Biosensors” (Drmosh et al., 2019). Enzyme-based sensors for hydrogen peroxide exhibit high sensitivity, selectivity, and a low detection limit. The operating temperature, relative humidity, and pH value, on the other hand, have a significant impact on the stability of enzyme-based hydrogen peroxide sensors (Asif et al., 2017). Furthermore, these sensors have a higher fabrication cost than non-enzyme–based hydrogen peroxide sensors. Due to the aforementioned limitations associated with enzyme-based hydrogen peroxide sensors, many attempts have been undertaken to produce non-enzymatic hydrogen peroxide sensors. As with enzyme-based sensors, the mechanism of sensing hydrogen peroxide non-enzymatically is entirely different from that of enzyme-based sensors, where electrocatalysts must fulfill several important criteria, including high electrocatalytic activity, large specific surfaces, strong conductivity, efficient electron transport from electrocatalysts to conductive substrates, stability, and repeatability (Khan et al., 2018; Aziz et al., 2022b). The noble metals (Au, Pd, and Pt) and their composites have been investigated as hydrogen peroxide sensor materials (Zhou et al., 2016; Jiao et al., 2017). Although noble metals are useful as hydrogen peroxide sensors, their high cost limits their practical application (Iftikhar et al., 2021). Many metal oxides have been investigated for their potential in fabrication and hydrogen peroxide sensor applications, including Co_3_O_4_, SnO_2_, Ag_2_O, Fe_2_O_3_, MnO_2_, NiOx, Cu_2_O, CoOx, and CuOx (Palanisamy et al., 2012; Xie et al., 2013; Bhati et al., 2018; Wang et al., 2018). Due to its abundance in nature, low fabrication costs, and favorable electrochemical and catalytic properties, ZnO is considered the best metal oxide for hydrogen peroxide sensors. Zinc oxide has been intensively explored due to its intriguing features, in addition to electrochemical sensors, photoelectric devices, gas sensors, and lithium-ion batteries (Ding et al., 2018). As a result of the superior catalytic properties of zinc oxide nanostructures compared to those of other metal oxide nanostructures, manufacture of low-cost hydrogen peroxide sensors based on ZnO nanostructures grown on conducting copper foil is possible (Asif et al., 2022b). Nanocombs, nanorods, nanotubes, and nanodisks, among all zinc oxide nanostructures, have more adsorption sites and a more significant surface area than nanoparticles and nanolayers. Recent research has focused on improving the electron transfer rate of ZnO nanostructures and the analyte sensing capability of the catalyst. Modifying ZnO nanostructures with Pt, Co, Ni, etc., nanoparticles increases their catalysis efficiency (Shen et al., 2020). In the preparation of active materials for sensors, several tedious steps must be completed, mostly by synthesizing nanomaterials individually and coating them on electrode surfaces. The reduction of active sites of the manufactured nanocomposite, spin coating, or drop-casting of the material on electrodes diminish electrocatalytic activity, resulting in low reproducibility and stability of the prepared electrode. As a result, high-performance nanostructures must be prepared by growing directly on the concerned electrode. This strategy will enhance the speed of electron transfer.

Thus, we present a facile chemical method of obtaining Cu-doped ZnO nanorods by modifying the electrode surface. The electrode was constructed and electrochemically analyzed in detail to obtain a large specific surface area, good electrical conductivity, and self-support structure to allow catalytic activities. As a result of this current investigation, the electrochemical sensor prepared expresses high electro-analytical performance and can be used as a sensing material for the next generation of sensors.

Using hydrothermal methods, we grew ZnO nanorods directly on FTO electrodes at low temperatures to identify an easy and feasible way of connecting nanoparticles to electrodes. In order to boost hydrogen peroxide sensing electrochemical activity, we prepared ZnO nanorods/FTO surfaces and deposited Cu nanoparticles on them (with larger surface areas and more active sites). Electrochemical activity for hydrogen peroxide sensing was increased as a result of the electron transfer. The morphological characterization of the copper–zinc oxide nanorods confirms that the Cu nanoparticles are attached to the zinc oxide nanorods in a uniform manner. With excellent stability, selectivity, repeatability, and reproducibility, Cu–ZnO nanorods exhibited excellent electrochemical results for hydrogen peroxide sensing. In addition, the modified electrochemical sensor yielded superior results for hydrogen peroxide detection in tap water and industrial water samples.

## Experimental Results

### Chemicals

Zinc acetate dihydrate (C_4_H_6_O_4_Zn.2H_2_O, 99%), zinc nitrate hexahydrate [Zn (NO_3_)_2_.6H_2_O, 98%], copper nitrate [Cu (NO_3_)_2_], hexamethylenetetramine (C_6_H_12_N_4_, 99%), ethanol (98%), hydrogen peroxide (H_2_O_2_), fluorine doped Tin-oxide (FTO), acetone (98%), alanine (Ala), dopamine (DA), citric acid (CA), penicillamine (Pen), glycine (Gly), ascorbic acid (AA), uric acid (UA), valine (Val), phenylalanine (Phe), and fructose, all analytical grade reagents, were purchased from Aladdin. The reagents were used in the analysis without any further purification. For the preparation of all solutions, deionized distilled water (18 MΩ cm) was used. The 0.1 mol/L PBS (pH = 7.0) electrolyte was used in all the experiments.

### Apparatus

Special instruments are used for morphological and chemical characterization. Scanning electron microscopy (SEM) measurements were taken with the JEOL, Japan (JSM-6390) and PHI-500 and Ulvac-Phi were used to perform X-ray photoelectron spectroscopy (XPS) (Japan). X-ray diffraction (XRD) measurements were taken with the D/MAX-3C (Japan), DPV (differential pulse voltammetry), EIS (electrochemical impedance spectroscopy), and CV (cyclic voltammetry), measurements were taken with the electrochemical work station (Gamry Reference 3000, Japan). As a working electrode, fluorine-doped tin-oxide (electrode area = 0.50 cm^2^ (active area = 0.30 cm^2^) was used. Pt wire was used as a counter electrode, and for the reference electrode, Ag/AgCl was used in the conventional three-electrode system.

### Modification of the Sensor

For the preparation of the sensor, FTO electrodes (geometric area = 0.50 cm^2^) were cleaned three times with an acetone-ethanol solution and then dried. Following the cleaning of the electrode surface, an insulating layer was placed, with only 0.30 cm^2^ of the electrode surface remaining uninsulated for modification and electrocatalytic activities. The working area can be adjusted using this insulating layer, as stated in the literature (Baruah et al., 2021). The ultra-thin layer of zinc oxide seeds (45–55 nm) on the electrode surface was deposited using an optimal procedure that included soaking the electrode (FTO) surface with zinc acetate dihydrate (0.005 M) solution, washing it with methanol, and drying it with Ar gas. The process is performed four to six times for preparing uniform seed growth on the electrode surface. The zinc acetate substance put on the electrode was then heated for 15 min at 300 (°C) degrees Celsius. The method is repeated two more times to ensure that the ZnO seeds are deposited in a uniform layer on the electrode surface. Hydrothermal ZnO nanorod development was achieved after uniformly coating ZnO seeds. The electrode was suspended inversely in a glass dish with a standard mixture of a fixed concentration of hexamethylenetetramine (0.06 M) and zinc nitrate dihydrate (0.04 M) at a fixed 90°C temperature for 2.5 h. When the reaction was finished, the zinc oxide nanorods generated on the specific electrode were cleaned to eradicate any impurities. The Cu nanoparticles were deposited on the ZnO nanorods grown in a prepared solution of 15 ml deionized distilled water, 0.5 ml methanol (as a reducing agent), and 0.03 g/ml of [Cu(NO_3_)_2_]. The mixture was transferred to an autoclave after 30 min outside and kept at 120°C for 80 min. The electrode was thoroughly cleaned with distilled water and absolute ethanol after the reaction was completed and allowed to cool to room temperature.

**SCHEME 1:**
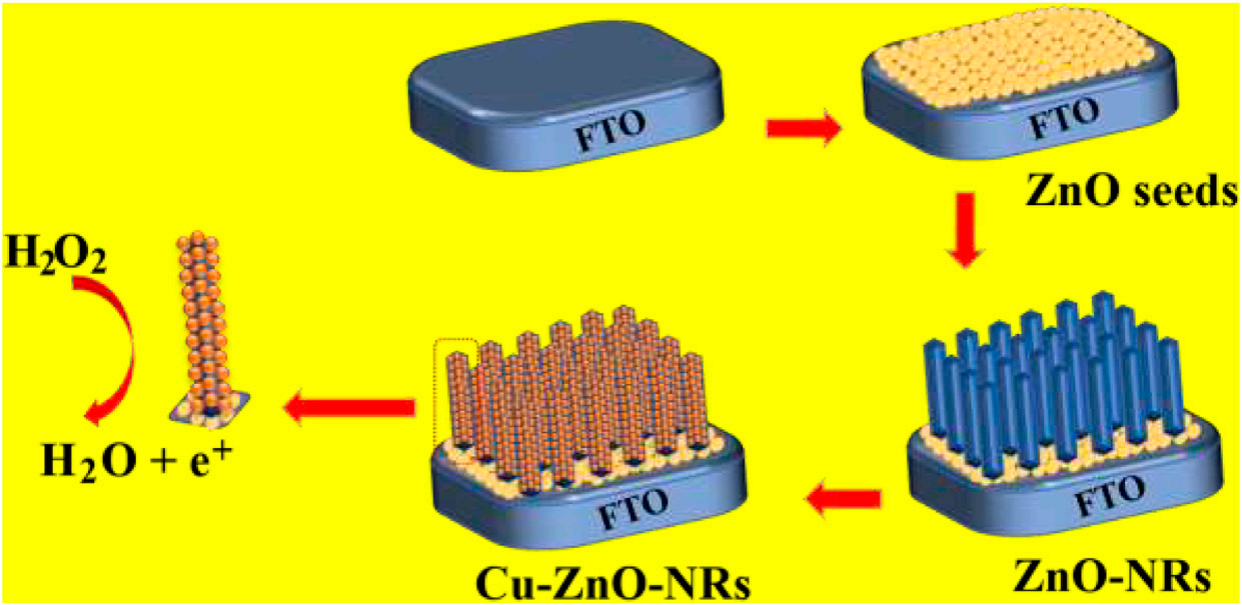
Synthesis and manufacturing of zinc oxide nanorods on the electrode surface, followed by the deposition of Cu nanoparticles for hydrogen peroxide detection, are depicted schematically.

Scheme 1 depicted the preparation and manufacturing of Cu–ZnO nanorods. We demonstrate the production and modification of an effective hydrogen peroxide sensor by hydrothermally growing zinc oxide nanorods directly on the concerned FTO electrode. The excellent hydrogen peroxide sensing results were obtained by depositing Cu nanoparticles on zinc oxide nanorods with enhanced surface area.

## Results and Discussion

### Physical and Chemical Characterization

**FIGURE 1:**
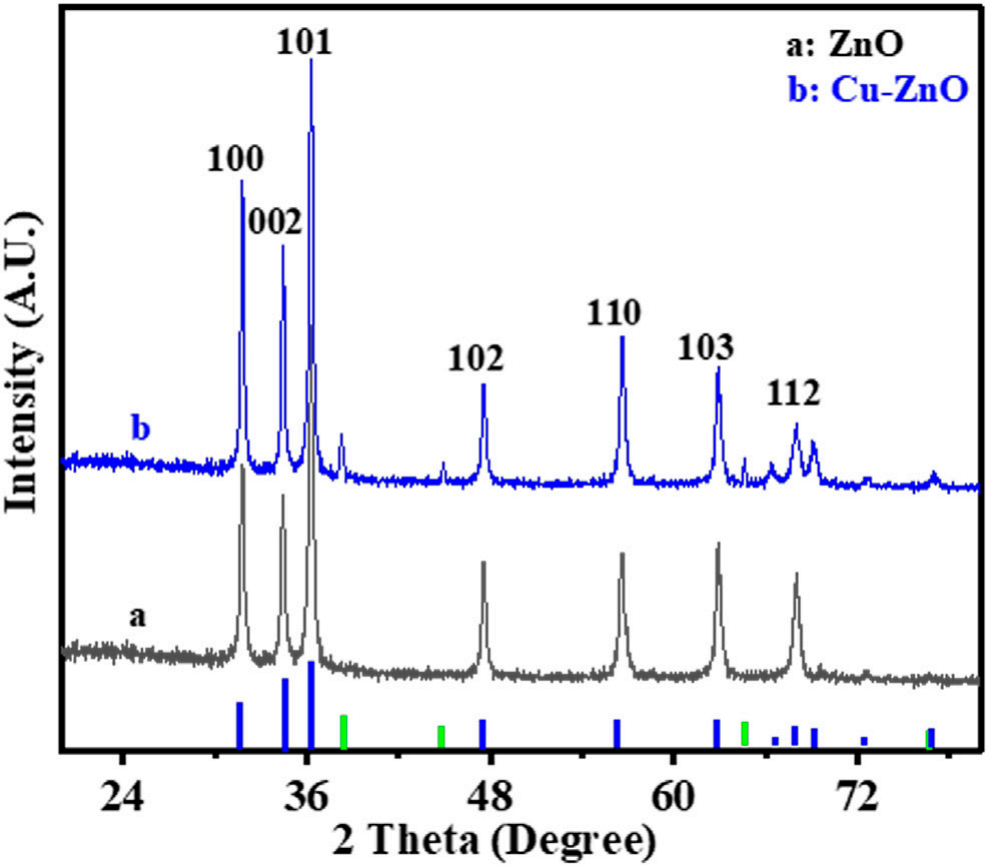
XRD spectral analysis of Cu-modified zinc oxide nanorods before (a) and after (b) deposition of Cu nanoparticles.

EIS, EDS, XPS, XRD, and SEM were used to characterize the morphology of the prepared and modified electrodes. Figure 1 depicts the zinc oxide nanostructure or morphology before and after the deposition of Cu nanoparticles. The XRD spectral analysis of manufactured zinc oxide nanorods before and after the synthesis of Cu nanoparticles is shown in Figure 1. The various peaks of zinc oxide nanorods indicated the hexagonal form of the nanorods. The Cu-ZnO nanorod spectra were also expressed after the modification with Cu nanoparticles, displaying all of the diffraction peaks and extra peaks of corresponding Cu nanoparticles. The hexagonal form of the prepared material was demonstrated by corresponding peaks at (100°), (101°), (102°), (103°), (002°), (110°), and (112°) planes, as indicated in literature (Kelly et al., 2019). The maximum peak intensity for the (101°) plane was measured at 2θ = 36.2°, with further peaks confirming the hexagonal form. Figure 1 shows the blue and green peaks, representing ZnO and Cu nanoparticles, respectively.

**FIGURE 2:**
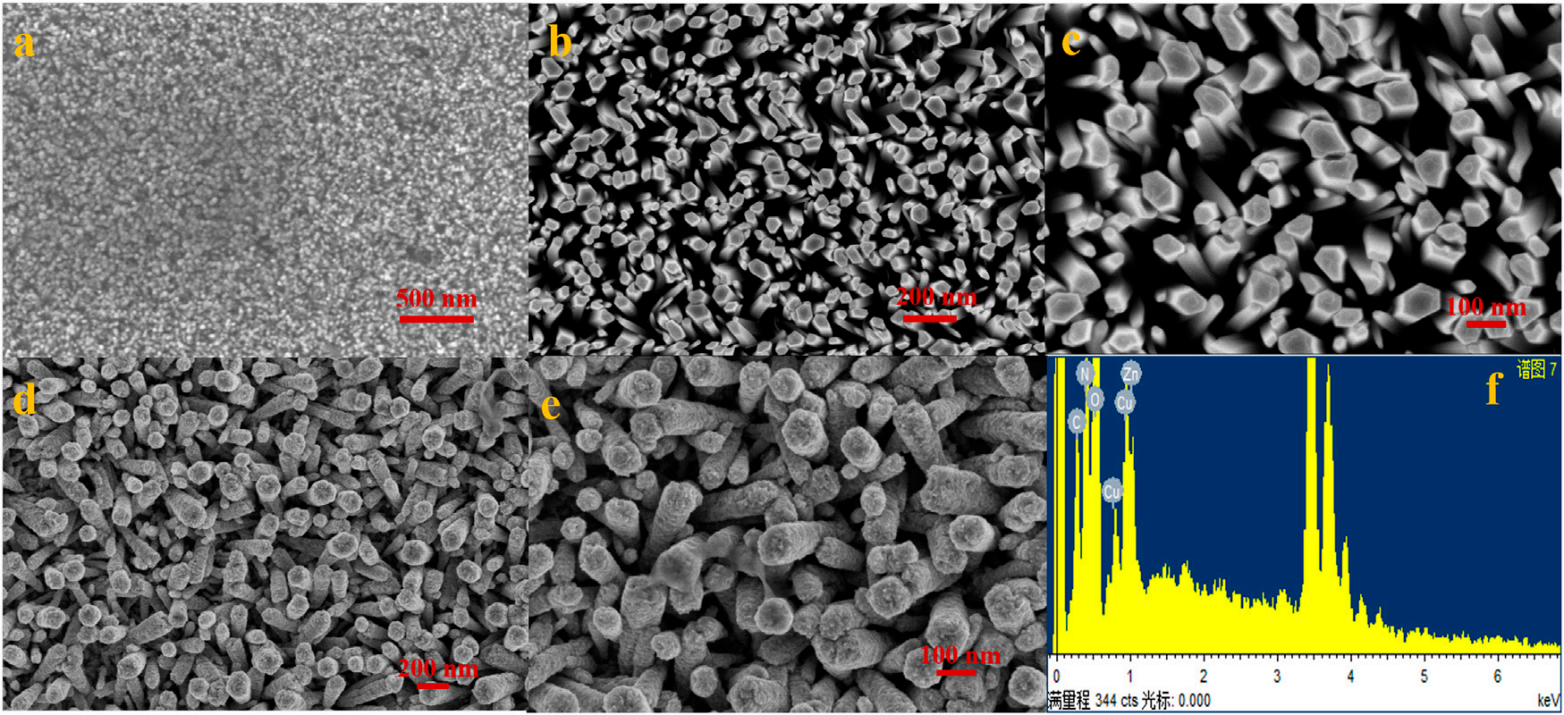
**(A)** Uniformly distributed ZnO nanoseeds on the substrate; **(B,C)** show the vertically developed zinc oxide nanorods on the concerned electrode surface; **(D,E)** shows the SEM images of fabricated copper-zinc oxide nanorods at low and high resolution; and **(F)** shows the EDS spectral analysis of copper–zinc oxide nanorods.

Figure 2 shows the results of SEM analysis of the manufactured electrode (A–E). Figure 2A shows an SEM image of ZnO nanoseeds that were equally scattered across the electrode surface. The zinc oxide nanorods were vertically generated and uniformly spread across the substrate’s surface, as shown in Figures 2B,C. The modified Cu-ZnO nanorods are shown in low- and high-resolution SEM images in Figures 2D,E. The Cu-Zn oxide nanorod rough surface was noticed when compared to the smooth surface of ZnO nanorods due to the production of Cu nanoparticles. Cu-ZnO nanorods had a diameter of about 100–150 nm. Figure 2F, which included the EDS analysis, was used to determine the composition of Cu-ZnO nanorods. EDS spectral analysis reveals the chemical composition of Cu, Zn, and O components in Cu-ZnO nanorods.

**FIGURE 3:**
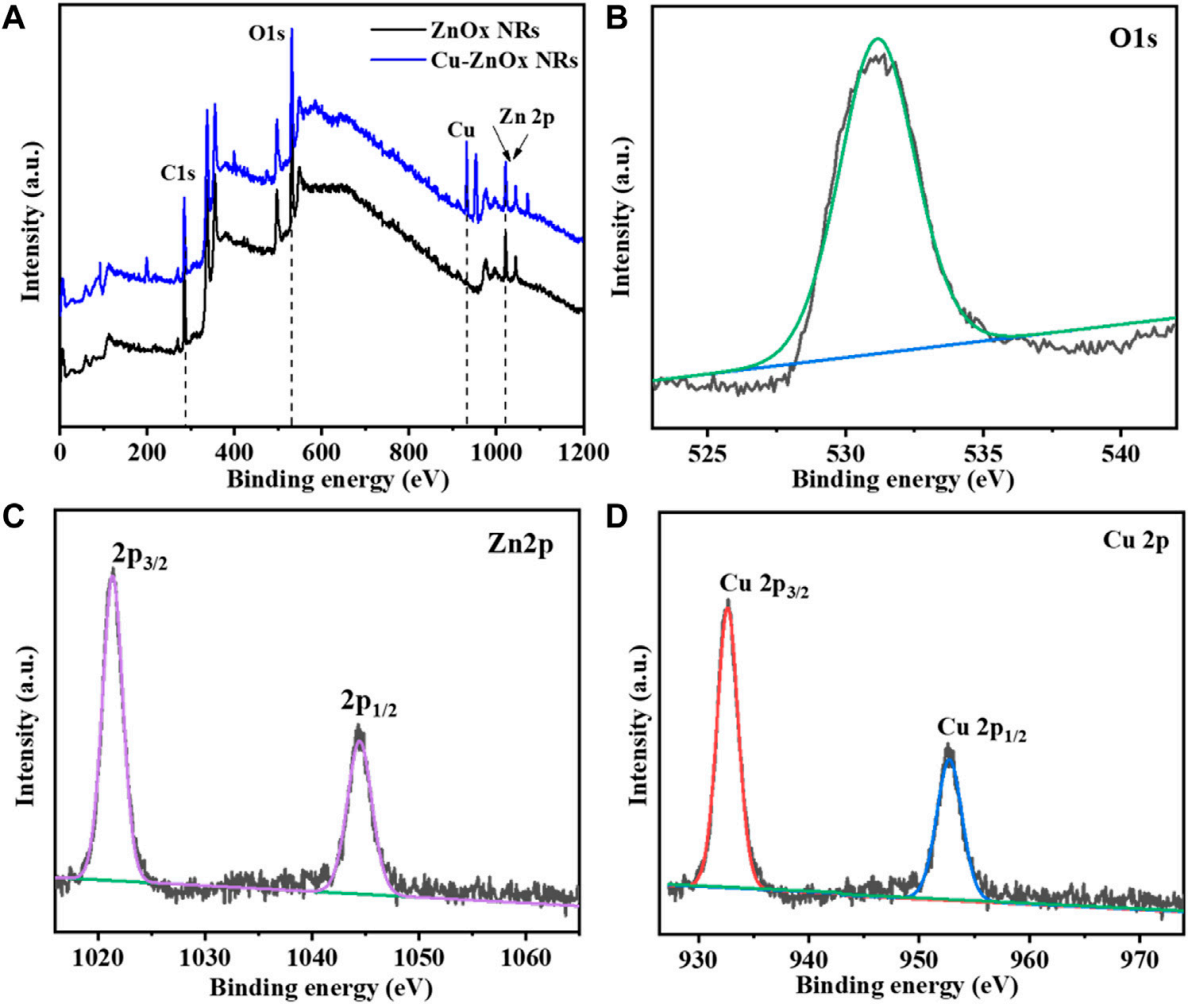
**(A)** Compare the XPS spectra of Cu-ZnO nanorods to the spectra of ZnO nanorods. It was also reported that the simplified peaks for oxygen **(B)** O1s, **(C)** zinc Zn 2p, and deposited material Cu 2p **(D)** elements with their magnified spectra were also mentioned.

XPS analysis was used to further characterize the chemical properties of Cu-ZnO nanorods and ZnO nanorods, as shown in Figure 3. Figure 3A shows a comparison of the entire XPS spectra of ZnO nanorods and Cu-ZnO nanorods. The whole scan reveals the presence of zinc, carbon, and oxygen atoms in zinc oxide nanorods. In contrast, the spectra of Cu-ZnO nanorods corroborated the presence of Cu, Zn, C, and O elements, indicating that Cu nanoparticles were successfully modified on ZnO nanorods. Figure 3B shows an expanded XPS spectrum of O 1s, which reveals a peak at 532.1 eV with a slightly higher binding capacity due to the modification of the surface of zinc oxide with copper (Al-Dairy et al., 2021). The Zn 2p peaks in Cu-ZnO nanorods were discovered to be at 1043.9 and 1021.2 eV, respectively, matching the Zn 2p_3/2_ and Zn 2p_1/2_, suggesting that the Zn valence state is 2^+^ (Kelly et al., 2019; Mishra et al., 2019). As illustrated in Figure 3, the addition of copper nanoparticles or fabrication of copper on zinc oxide nanorods results in binding energy shifts of 932.5 and 952.3 eV for Cu 2p_3/2_ and 2p_1/2_, respectively (d). It was also shown that when compared to bulk Cu (953 eV), Cu 2p_3/2_ had a binding energy shift of 0.51 eV (Awais et al., 2021a; Fariba Beigmoradi and Hadi Beitollahi, 2021). The binding energy shifting in the negative direction was observed due to electron transfer from zinc oxide to copper, which could have been produced by the intensive attraction of copper nanoparticles with zinc oxide (Chen et al., 2018).

### Electrochemical Studies

**FIGURE 4:**
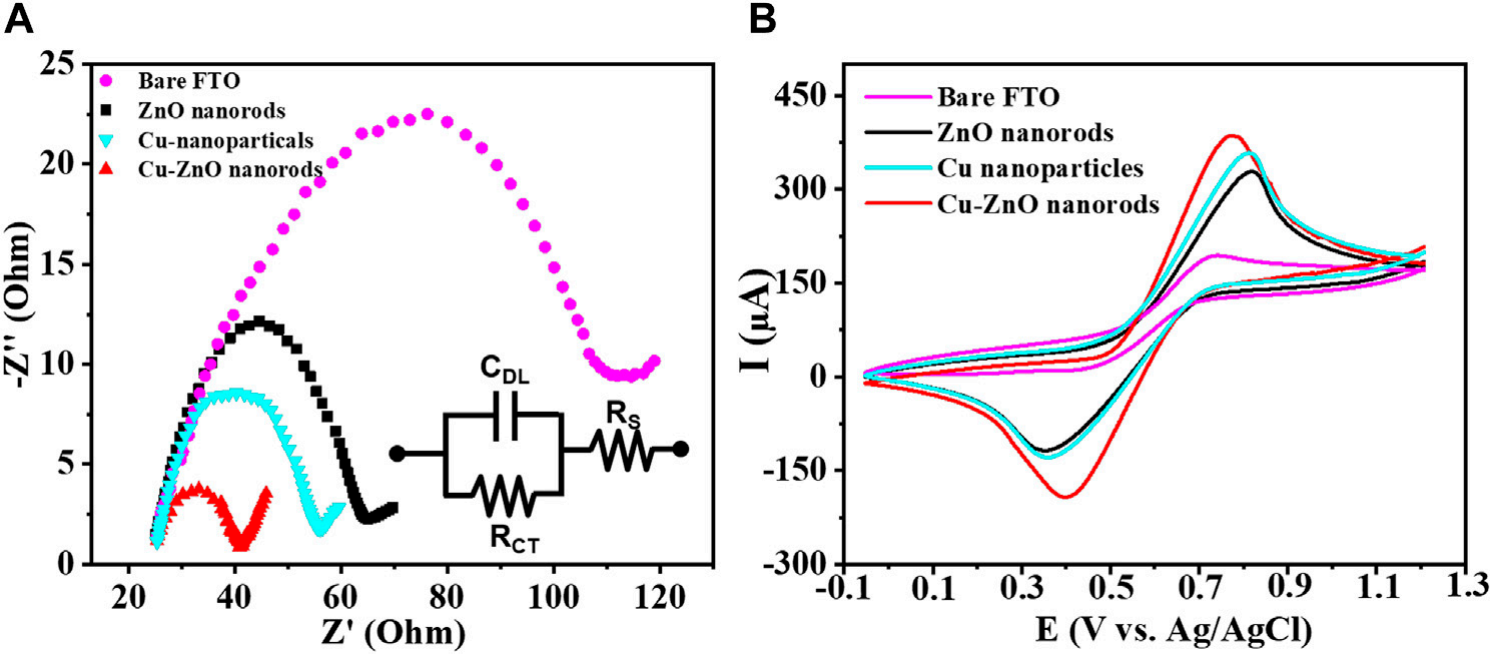
**(A)** Resistance capacity of Cu-ZnO nanorods, zinc oxide nanorods, Cu nanoparticles, and blank FTO, was best described by plotting Nyquist semicircle in standard 0.1 mol/L KCl containing a 0.1 M K_4_ [Fe(CN)_6_] and K_3_ [Fe(CN)_6_] solution. **(B)** CV results of blank FTO with further modified electrodes zinc oxide nanorods, Cu nanoparticles, and Cu-ZnO nanorods were mentioned in standard KCl containing K_4_ [Fe(CN)_6_] and K_3_ [Fe(CN)_6_] solution.

Some analysis was carried out to better understand the electrochemical characteristics of the produced nanostructure. Figure 4A expressed the EIS spectral analysis of each electrode in standard potassium chloride solution containing K_4_ [Fe (CN)_6_] and K_3_ [Fe (CN)_6_] solution (for blank FTO, zinc oxide nanorods, Cu nanoparticles, and Cu-ZnO nanorods). The impedance findings were evaluated using the Rct values (as mentioned in the insert of Figure 4A). The Rct value of blank FTO (purple color) is the highest, with a value of Rct = 92, compared to ZnO nanorods (black color) with Rct = 40.5 and copper nanoparticles (light blue color) with Rct = 32.7. When compared to bare FTO, the Rct values reduced, indicating an enhanced electron transfer rate. We used prepared ZnO nanorods with Cu nanoparticle deposition in order to select the more competent electrode. The Rct value was further decreased after copper nanoparticle modification on ZnO nanorods, and Cu-ZnO nanorods (red color) exhibit low resistance when compared to all other electrodes, with Rct = 15.8. The fabricated copper–zinc oxide nanorod electrode exhibits the lowest resistance rate for electron transfer when related to other modified electrodes. For the additional electrochemical study, a Cu-ZnO nanorod electrode was used. In 0.1 mol/L KCl solution containing 0.1 mol/L K_3_ [Fe (CN)_6_]/K_4_ [Fe (CN)_6_], cyclic voltammetry analysis was conducted to investigate the electrochemical reaction of blank FTO with different modified electrodes such as zinc oxide nanorods, Cu nanoparticles, and Cu–ZnO nanorods. Figure 4B illustrates the current responses for oxidation peaks determined from cyclic voltammetry analysis for blank FTO, zinc oxide nanorods, Cu nanoparticles, and Cu–ZnO nanorod electrodes. The Cu–ZnO nanorod electrode had the largest redox peak current, while other ZnO nanorods, Cu nanoparticles, and blank FTO electrodes had less current value. When modified ZnO nanorod, Cu nanoparticle, and Cu-ZnO nanorod electrode comparison was carried out in CV experiments, the blank FTO had the lowest redox potential current. From the CV study, it was concluded that Cu-ZnO nanorods were a good contender for detecting the analyte in the study.

**FIGURE 5:**
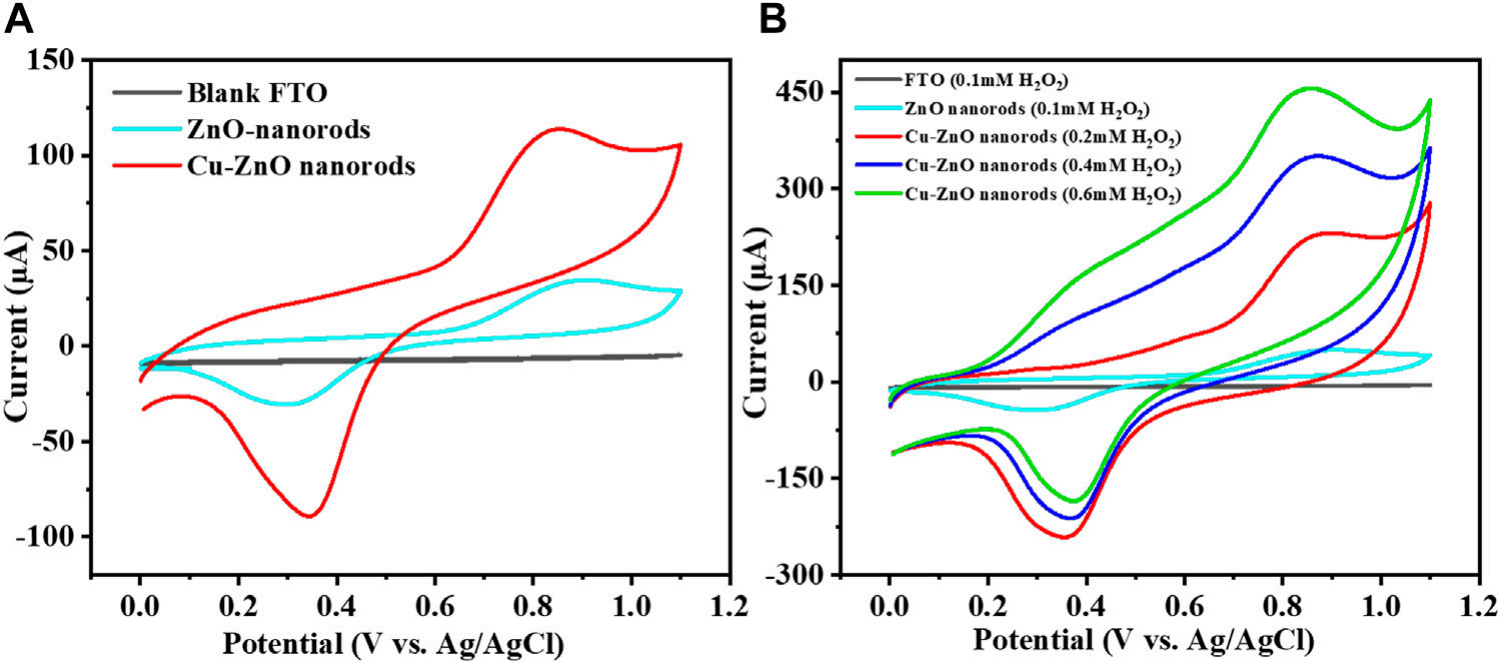
**(A)** Expressed that at a fixed scan rate of 50 mVs^−1^, and the CV curve of zinc oxide nanorods, Cu-ZnO NRs, and blank FTO in 0.1 mol/L phosphate buffer containing 0.2 mM hydrogen peroxide was mentioned. **(B)** Shows the various hydrogen peroxide concentrations, the CV curves of blank FTO, zinc oxide nanorods, and produced Cu-ZnO nanorods are compared.

The simplest and most suitable method to investigate the electrochemical performance is the cyclic voltammetry method. CV results of different electrodes were analyzed in an electrochemical cell with specific potential ranges from 0 to +1.15 V to select the best potential electrode. Figure 5A shows the CV results at a fixed scan rate of 50 mVs^−1^ of produced Cu–ZnO nanorods in 0.1 mol/L phosphate buffer electrolyte containing 0.2 mM hydrogen peroxide. Fabricated Cu–ZnO nanorods were shown to have good current activity. As compared to CVs of various electrodes in 0.2 mM, hydrogen peroxide was also conducted, demonstrating a higher oxidation current. As shown in Figure 5B, the modified Cu–ZnO nanorods have outstanding catalytic properties when compared to those of blank FTO and zinc oxide nanorods. Modified Cu-ZnO nanorods exhibited a well-defined oxidation peak at +0.8 V in 0.2 mM hydrogen peroxide. The results of fabricated Cu-ZnO nanorods suggested that the electrode might be used for hydrogen peroxide electro-oxidation. Figure 5B clearly shows that as the hydrogen peroxide concentration rises, the current increases as well, indicating that the electrode has potential for detecting hydrogen peroxide. Supplementary Figure S1 depicted a schematic example of hydrogen peroxide sensing. In the absence and presence of 0.2 mM hydrogen peroxide, CV measurements of a fabricated copper–zinc oxide nanorod electrode in 0.1 mol/L phosphate electrolyte at various scan speeds from 20 to 200 mVs^−1^ were mentioned (Supplementary Figures S1A,B). The current response increased in the presence of hydrogen peroxide, indicating the catalytic activity of the fabricated material, according to CV data.

### Detection Results of Modified Cu-ZnO Nanorod Electrode

**FIGURE 6:**
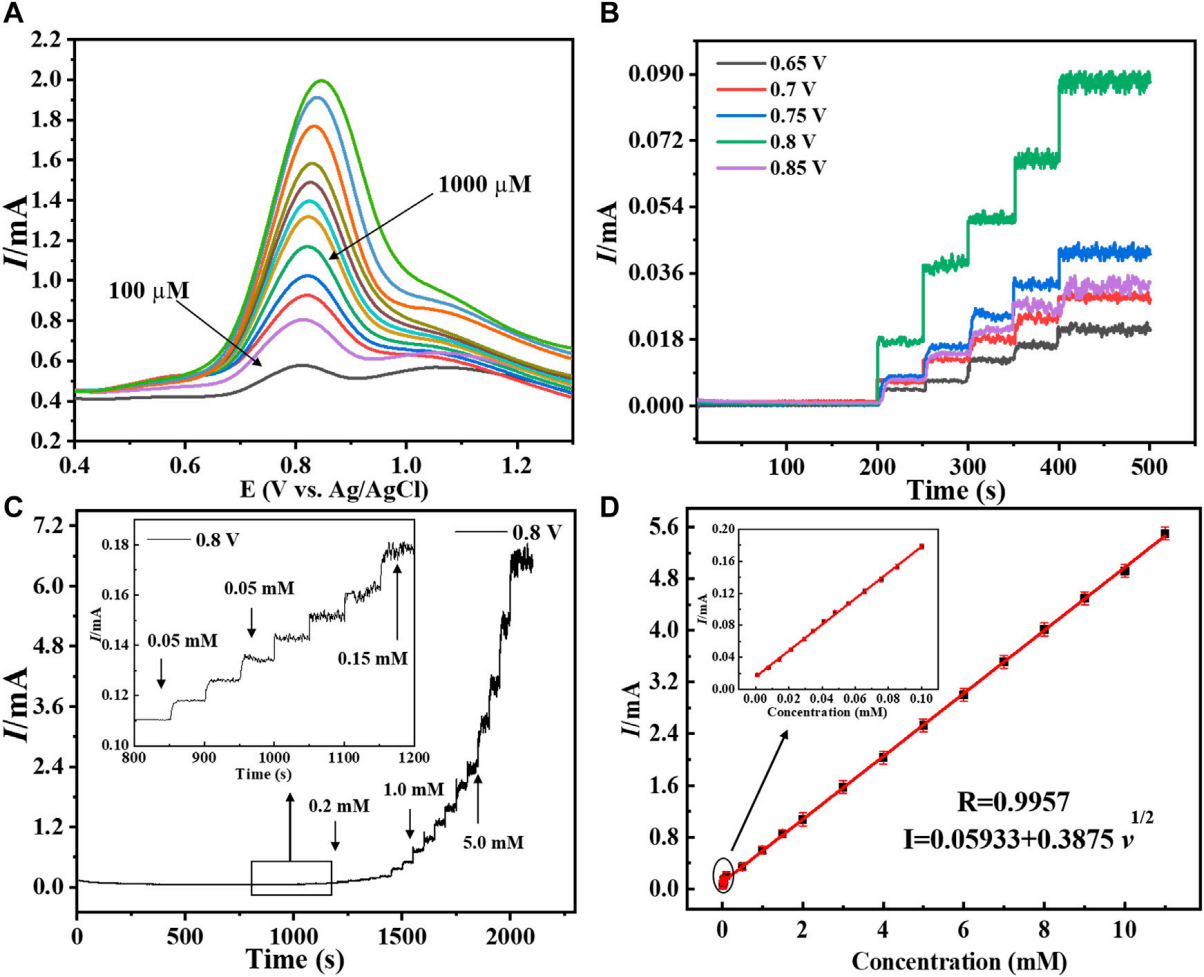
**(A)** Depicted the DPV graph of Cu-ZnO nanorods at various hydrogen peroxide concentrations. **(B)** Amperometric response of copper-zinc oxide nanorods in 0.1 mol/L phosphate electrolyte with varied potentials ranging from (0.65–0.85 V) was discussed. The I-T graph of Cu-ZnO nanorods, with various hydrogen peroxide concentrations, is shown at a specific potential of +0.8 V **(C)**. The insert shows a magnified I-T graph of Cu-ZnO nanorods. For Cu-ZnO nanorods, the corresponding linear graph of current vs. hydrogen peroxide concentration is displayed in **(D)**. Insert mentioned the magnified linear graph.

The modified electrode’s for hydrogen peroxide sensing activity was tested in order to exhibit analytical parameters such as linear range, response time, detection limit, and sensitivity. Figure 6A, shows the sensing performance of the prepared electrode to evaluate its sensing activity using DPV. It was discovered that as the concentration increased, the current increased as well, implying that the fabricated copper–zinc oxide nanorod catalyst is suited for hydrogen peroxide sensing. In addition, as indicated in the literature (Hu et al., 2022), amperometric studies with modified Cu-ZnO nanorods were conducted at various potential ranges between +0.65 and +0.85 V to evaluate the suitable potential for hydrogen peroxide detection. With continued addition of standard hydrogen peroxide concentrations, at a fixed scan rate of 50 mVs^−1^, the amperometric response in 0.1 mol/L phosphate electrolyte was evaluated at various applied potentials. Figure 6B demonstrates that when compared to other applied potentials, the Cu-ZnO nanorods were chosen for long-term current-time study because it has the highest potential at +0.8 V. The Randles–Sevcik equation was used to calculate the active surface area of the sensing electrode, and the CV technique is used to investigate it in a deoxidized mixture containing standard potassium chloride solution containing K_4_ [Fe (CN)_6_] and K_3_ [Fe (CN)_6_] solution, at various scan rates, as shown in Supplementary Figure S2. The active surface areas of ZnO nanorods (0.328 cm^2^), Cu nanoparticles (0.319 cm^2^), and Cu-ZnO nanorods (0.369 cm^2^) were observed. After carefully observing the surface area, the optimum pH of the electrolyte was also obtained for better sensing activity. To evaluate the optimum pH of the electrolyte, the amperometric analysis was conducted at a fixed scan rate of 50 mVs^−1^ in 0.1 mol/L PBS (at different pH) electrolyte at a fixed current of +0.8 V, and their linear graph is expressed in Supplementary Figure S3. The linear graph of different pH values from pH 6.0 to pH 8.0 was mentioned. The results show that the optimum pH of the electrolyte which shows maximum current or sensitivity is at pH 7.0. So, pH 7.0 is considered optimum pH for concerning materials and is used in all amperometric analyses.

The long I-T analysis was conducted at a fixed scan rate of 50 mVs^−1^ in 0.1 mol/L PBS (pH = 7.0) electrolyte at a fixed current of +0.8 V. The current response developed slowly at lower concentrations but rapidly at higher concentrations, as shown in Figure 6C. The linear range of 0.001–11 mM of Cu-ZnO nanorod modified electrode for hydrogen peroxide sensing was observed. For comparison, the current-time responses of Cu nanoparticles@FTO and ZnO nanorods@FTO electrodes were also examined. According to the findings, Cu-ZnO nanorods have substantially higher responsiveness than other electrodes. The enlarged current response of all electrodes at lower concentrations is shown in the insert. With increasing concentration, the current response increases linearly. The amperometric studies for linearity for hydrogen peroxide current vs. concentration are shown in Figure 6D. The limit of detection was found to be 0.16 M, and the coefficient of correlation (R_2_) value was observed to be R_2_ = 0.9957. Furthermore, the current was shown to increase linearly with the increase in hydrogen peroxide concentration from 0.001 to 11 mM. The hydrogen peroxide detection sensitivity of a modified Cu-ZnO nanorod electrode was measured to be 3415 μAmM^−1^cm^−2^ as mentioned in Table 1. In Table 1, we compare the detecting activity of our modified Cu-ZnO nanorod electrode to that of various previously published hydrogen peroxide electrodes. Table 1 demonstrates that the fabricated Cu-ZnO nanorods have a significantly higher sensing activity of 3415 μAmM^−1^cm^−2^ than previously reported electrodes and a lower LOD and a much wider linear range. The Cu-ZnO nanorod sensor that was prepared has a large linear range and a good reaction time. Chronoamperometry was used to determine the reaction time of the manufactured electrode. The chronoamperogram revealed that Cu-ZnO nanorods have a good reaction time. The reaction time of Cu-ZnO nanorods was 1.49 s, which is decent when compared to that of previous sensors. In addition, our fabricated electrode performed better in terms of sensing since it was grown directly on ZnO nanorods with a larger surface area for Cu deposition. The modified Cu-ZnO nanorod electrode showed a high electron transfer rate in an electrochemical method for hydrogen peroxide oxidation. The use of a hydrothermal approach to modify a non-enzymatic sensor results in an outstanding nanostructure with a cost-effective, stable, and larger surface area for hydrogen peroxide sensor modification.

**TABLE 1:** Comparison of different electrodes for hydrogen peroxide sensing.

Electrode	Sensitivity (μA mM^−1^ cm^−2^)	Linear range (mM)	Lower detection limit (μM)	Reference
CuO nanofibers	431.3	0.006–2.5	0.8	Hussain et al. (2016)
ZnO composite	2961.7	Up to 8.45	0.4	Kannadasan et al. (2014)
CuO nanosphere	404.53	0–2.55	1	Li et al. (2019)
ZnO nanocombs	1533	0.02–4.5	20	Rashed et al. (2021)
GOD/ZnO	30	0.001–0.76	0.7	Al-Hardan et al. (2016)
ZnO-Ag	3.85	0.015–6.5	1.5	Shahkhatuni et al. (2019)
ZnO-Ni	61.78	0.5–8	2.5	Xu et al. (2020)
ZnO-Co	13.3	0–4	20	Xie et al. (2013)
ZnO nanocrystal	1091.1	0.6–1.4	0.22	Zang et al. (2007)
Cu-ZnO nanorods	3415	0.001–11	0.16	This work

### Reproducibility, Reusability, Anti-Interference Activity, and Stability

**FIGURE 7:**
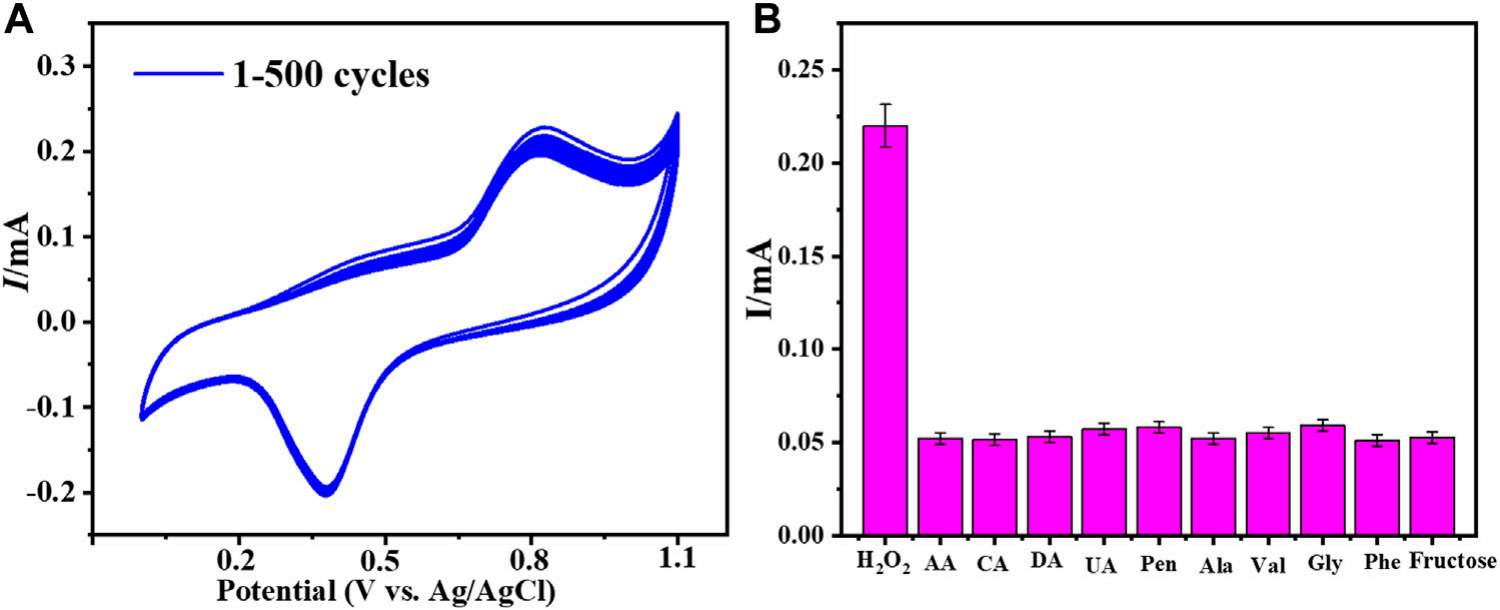
**(A)** Cu-ZnO nanorod stability was evaluated for 500 cycles in 0.2 mM hydrogen peroxide in phosphate electrolyte at a fixed scan rate of 50 mVs^−1^. **(B)** Anti-interference results for all probable interfering compounds such as fructose, Phe, Ala, Gly, Val, DA, CA, UA, AA, and Pen were expressed.

The fabricated electrode’s reusability and repeatability were assessed in order to assess its performance. To assess the performance of Cu–ZnO nanorods, a reproducibility and reusability test was carried out on ten samples, each of which contained the same amount of hydrogen peroxide. The CV response was evaluated in 0.1 mol L^−1^ PBS (pH = 7.0) vs. Ag/AgCl at a fixed scan rate of 50 mVs^−1^. After numerous uses, the produced electrode retains up to 98.2 percent of its sensing ability; this indicates that the produced electrode is repeatable and reusable. The RSD was estimated to be approximately 3.83 %. CV analysis responding in 0.2 mM hydrogen peroxide at a fixed scan rate of 50 mVs^−1^ in phosphate buffer was used to examine the stability of fabricated Cu-ZnO nanorods. The stability graph of Cu-ZnO nanorods during 500 cycles is shown in Figure 7A. The electrode’s stability is improved by nanorods generated directly on the electrode surface. The long-term durability of the manufactured electrode was also examined after 15 days, and there were few changes; as illustrated in Supplementary Figure S4, the modified electrode remained stable for a long time. The ability to distinguish between the target analyte and interfering species with similar electrocatalytic activity is one of the most significant analytical skills of electrochemical sensors. Major interferences which can impact hydrogen peroxide sensing include fructose, citric acid (CA), ascorbic acid (AA), dopamine (DA), and uric acid (UA). The anti-interference ability of the fabricated electrode for hydrogen peroxide detection was tested using aline (Val), fructose, alanine (Ala), citric acid (CA), phenylalanine (Phe), glycine (Gly), ascorbic acid (AA), dopamine (DA), uric acid (UA), and penicillamine (Pen). The amperometric response was evaluated at a fixed scan rate of 50 mVs^−1^ in the presence of 0.2 mM hydrogen peroxide in 0.1 mol/L phosphate electrolyte, by adding 0.1 mM of each interfering chemical. Figure 7B shows the result of those interfering substances as a graphical form. Fabricated Cu-ZnO nanorods results expressed that it has no obvious response to all other interfering substances, showing excellent selectivity for amperometric hydrogen peroxide detection. Due to the nature of the material, the Cu-ZnO nanorods display extremely few interferences at relatively high potential.

### Hydrogen Peroxide Detection in Water Samples

**FIGURE 8:**
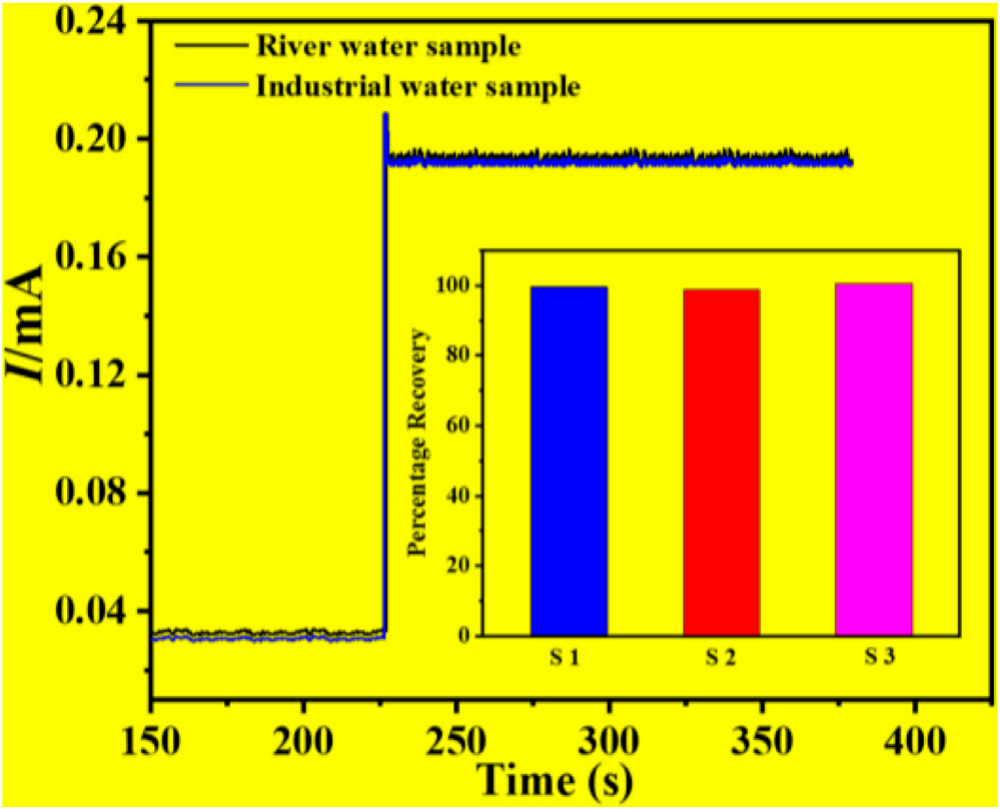
Percentage of recovery results in an actual water sample test of a water sample and an industrial water sample, with a histogram graph was mentioned.

Some studies were carried out in order to determine the use of the constructed Cu-ZnO nanorod electrode for hydrogen peroxide detection. The I-T graphical result of Cu-ZnO nanorods was obtained by adding 2 ml of the water sample or industrial water sample into 8 ml of 0.1 mol/L phosphate at a fixed current of +0.8 V as shown in Figure 8. The results of water samples were compared and mentioned. The findings of many samples were gathered and displayed in a histogram graph in Figure 8. When compared to analytically measured values, the results demonstrated that hydrogen peroxide detection in water samples was excellent, having various kinds of substances present in the water sample (Bhati et al., 2018), and the water sample has a 2.5 percent lower detection activity for hydrogen peroxide. The findings suggested that the manufactured Cu-ZnO nanorod sensor might be used in real-environment water samples.

## Conclusion

Finally, vertically growing copper–zinc oxide nanorods on the electrode surface were successfully constructed for excellent hydrogen peroxide detection. Physiochemical and electrochemical approaches were used to characterize the Cu–ZnO nanorods. The hexagonal Cu–ZnO nanorods had a large surface area and excellent electrochemical properties for hydrogen peroxide detection. The non-enzymatic electrochemical sensor that was created has a large linear range, a low detection limit, strong selectivity, and a good response toward hydrogen peroxide. The reaction time, stability, repeatability, and reproducibility of the Cu–ZnO nanorods were all excellent. Furthermore, the hydrogen peroxide sensing activity in a real water sample demonstrated the sensor’s excellent results with a recovery percentage of 98%. The fabricated copper–zinc oxide nanorod electrode outperformed ZnO nanorods, copper nanoparticles, and other electrodes in terms of electrochemical performance. The manufactured Cu-ZnO nanorods proved to be a viable candidate for hydrogen peroxide sensing in the actual world with excellent real-environment detection of the concerned analyte.

## Data Availability

The original contributions presented in the study are included in the article/Supplementary Material; further inquiries can be directed to the corresponding authors.
