# Supplementary material for: Novel Synthesis of Sensitive Cu-ZnO Nanorod–Based Sensor for Hydrogen Peroxide Sensing
Source: Front Chem. 2022 Jul 6;10:932985. doi: 10.3389/fchem.2022.932985 (PMC9298554; doi:10.3389/fchem.2022.932985)
Supplement: Supplementary file 1 [file DataSheet1.docx]

**Supplementary Information**

**Novel Synthesis of Sensitive Cu-ZnO Nanorods based sensor for Hydrogen Peroxide Sensing**


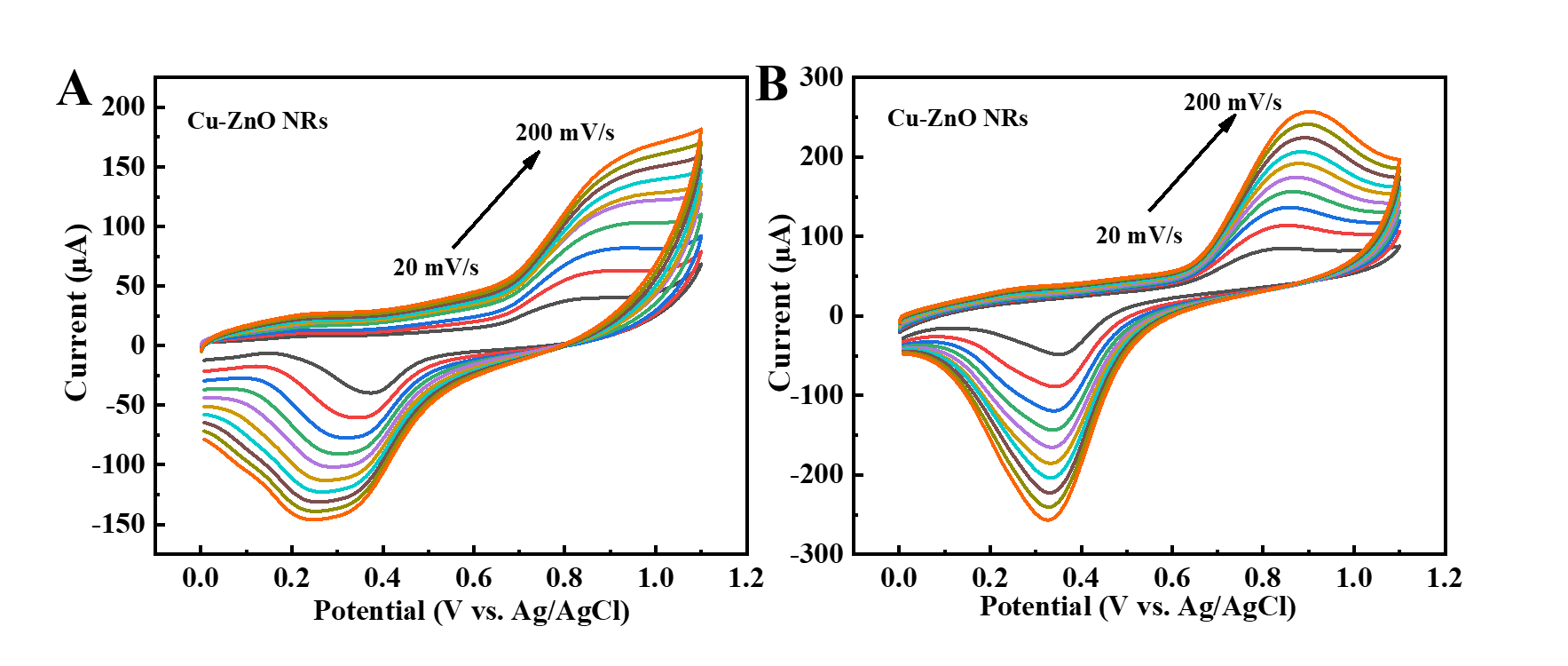


**Supplementary image 1.** In the absence and presence of 0.2 mM H_2_O_2_, the cyclic voltammetry curve of modified copper zinc oxide nanorods was described at varied scan rates of 20 to 200 mVs^-1^ in 0.1 mol/L PBS (pH=7.0).


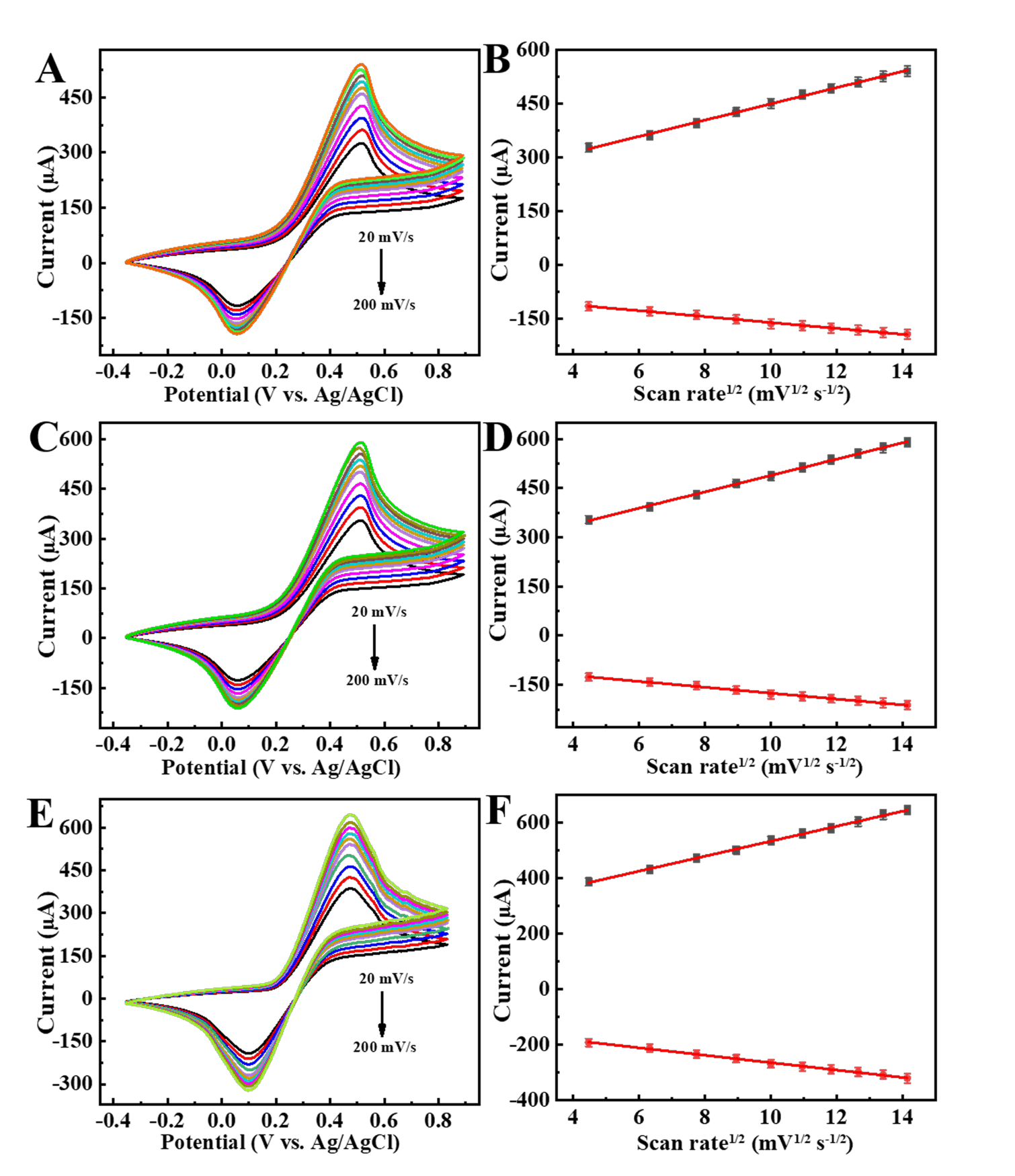


**Supplementary image 2.** At varied scan rates from 20 to 200 mVs^-1^, the CV for ZnO nanorods (a), copper nanoparticles (c), and Cu-ZnO nanorods (e) in a mixture containing 0.1 mol/L K_3_[Fe(CN)_6_]/K_4_[Fe(CN)_6_] (mass ratio of 1:1) and 0.1 mol/L KCl without O_2_. In the deoxidized mixture including 0.1 mol/L K_3_[Fe(CN)_6_]/K_4_[Fe(CN)_6_] (mass ratio of 1:1) and 0.1 mol/L KCl, the oxidation reduction peak linear relationship between current and scan rate1/2 for ZnO nanorods (b), copper nanoparticles (d), and Cu-ZnO nanorods (f) was mentioned.


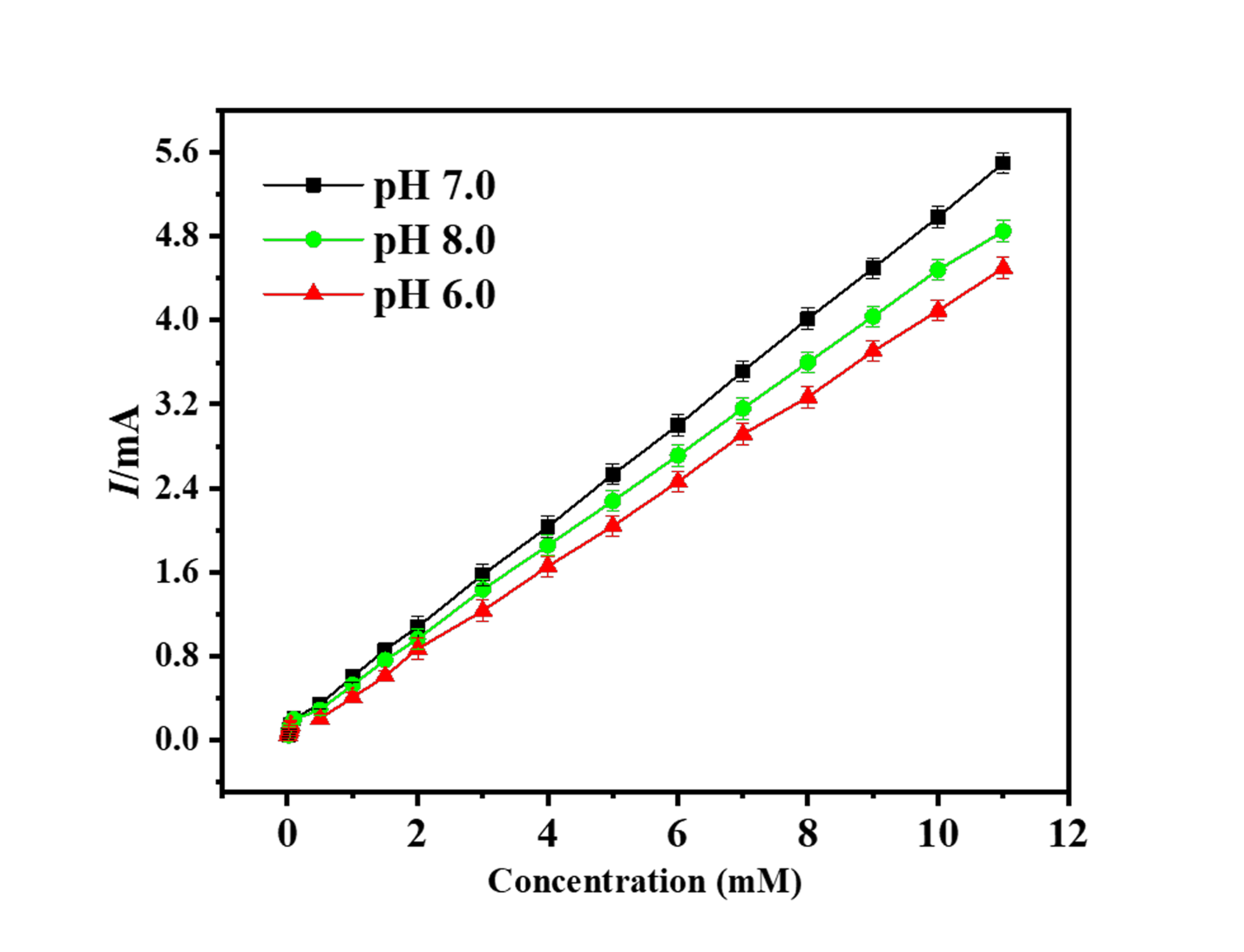


**Supplementary Image 3.** The sensing activity of concern material (Cu-ZnO nanorods) at different pH values was perform and their linear graph was expressed with different pH values from pH 6 to pH 8 at a fixed scan rate of 50 mVs^-1^.


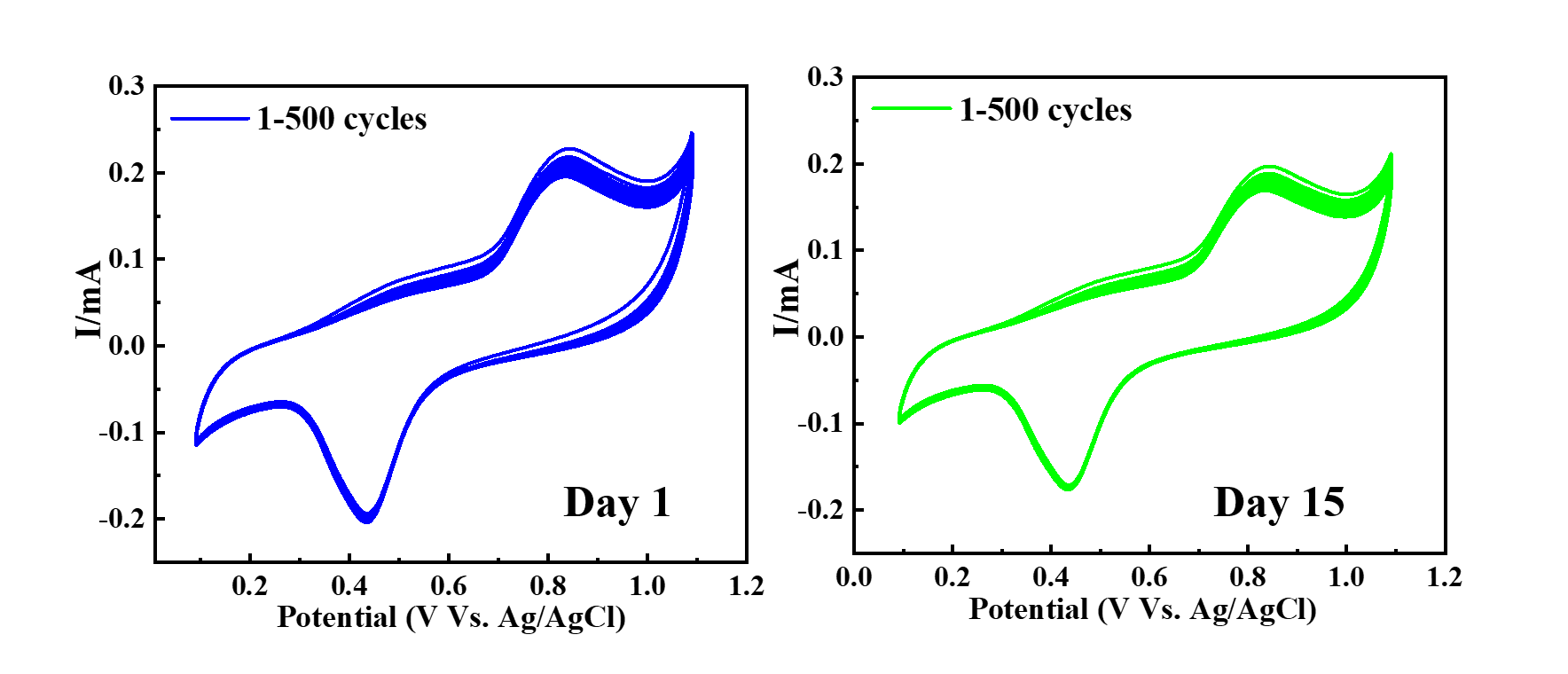


**Supplementary image 4.** Cu-ZnO nanorods were tested for long-term stability at a fixed scan rate of 50 mVs^-1^ in 0.1 mol/L PBS (pH=7.0) in the presence of 0.2 mM H_2_O_2_.
